# Correlation of serum N-Acetylneuraminic acid with the risk and prognosis of acute coronary syndrome: a prospective cohort study

**DOI:** 10.1186/s12872-020-01690-z

**Published:** 2020-09-10

**Authors:** Miao-Nan Li, Shao-Huan Qian, Zhuo-Ya Yao, Sheng-Ping Ming, Xiao-Jun Shi, Ping-Fang Kang, Ning-Ru Zhang, Xiao-Jing Wang, Da-Sheng Gao, Qing Gao, Heng Zhang, Hong-Ju Wang

**Affiliations:** 1 Anhui Provincial Hospital, Cheeloo College of Medicine, Shandong University, Jinan, Shandong 250021 China; 2 Department of Cardiovascular Disease, The First Affiliated Hospital of Bengbu Medical College, 287 Zhihuai Road, Bengbu, 233000 Anhui China; 3 Anhui Clinical and Preclinical Key Laboratory of Respiratory Disease, Bengbu, Anhui China; 4 Anhui Provincial Key Laboratory of Computational Medicine and Intelligent Health, Bengbu, Anhui China

**Keywords:** N-Acetylneuraminic acid, Acute coronary syndrome, Myocardial infarction, GRACE score, Major adverse cardiovascular events

## Abstract

**Background:**

N-acetylneuraminic acid (Neu5Ac) is a functional metabolite involved in coronary artery disease (CAD). We aimed to evaluate the relationship between serum Neu5Ac and the risk and prognosis of acute coronary syndrome (ACS) in a real-world prospective study.

**Methods:**

Patients with suspected ACS who underwent coronary angiography were included. Serum Neu5Ac was measured at admission. Coronary lesion severity was evaluated by Gensini Score. GRACE risk stratification was performed at admission. Major adverse cardiac events (MACEs) were recorded during follow-up.

**Results:**

A total of 766 patients, including 537 with unstable angina (UAP), 100 with myocardial infarction (MI), and 129 without CAD were included. The circulating Neu5Ac level was significantly higher in patients with MI (median [1QR]: 297[220, 374] ng/ml) than in those with UAP (227 [114, 312] ng/ml) or without CAD (207 [114, 276] ng/ml; both *p* < 0.001). Serum level of Neu5Ac was positively correlated with age, hypertension, serum uric acid, creatinine, MB isoform of creatine kinase (CK-MB), and Gensini score (all *p* < 0.05). Receiver operating characteristic curve analysis showed that a higher serum Neu5Ac was potentially associated with MI and high-risk GRACE stratification in ACS patients. Logistic analysis identified only elevated serum Neu5Ac as an independent predictor of MACEs in these patients (odds ratio [OR]: 1.003, 95% confidence interval [CI]: 1.002–1.005, *p* < 0.001).

**Conclusions:**

Serum Neu5Ac is associated with myocardial injury, GRACE risk category, and prognosis in ACS patients.

## Background

Acute coronary syndrome (ACS) refers to a severe subtype of coronary artery disease (CAD) that is characterized by the acute rupture of vulnerable atherosclerotic plaques in the coronary arteries and subsequent complete or incomplete obstruction of coronary blood flow due to thrombosis formation [1, 2]. Patients with ACS often suffer from acute symptoms of chest pain or dyspnea, which are mainly caused by acute myocardial ischemia [3, 4]. Currently, ACS can be classified as ST-segment elevation myocardial infarction (STEMI) and non ST-segment elevation ACS (NSTE-ACS) according to the dynamic changes in the ST segment on electrocardiography (ECG) [5–7]. Although substantial advancements have been achieved in the diagnosis and treatment of ACS in recent decades, including percutaneous coronary intervention (PCI), patients with ACS remain at great risk for the development of adverse outcomes, including malignant arrhythmia, cardiac shock, heart rupture, and death [8]. Therefore, studies of the key molecular pathways involved in the pathogenesis and progression of ACS are still of fundamental clinical importance for improving risk stratification and targeted treatment in these patients [9].

Accumulating evidence suggests that both genetic factors and environmental factors contribute to the pathogenesis of ACS [10]. Interactions between genetic and environmental factors may change various metabolic pathways during atherosclerosis development, leading to changes in the level of metabolites of the myocardium [10]. Therefore, detection of some metabolites may be helpful for clarifying the pathophysiological mechanism of the disease [11]. N-acetylneuraminic acid (Neu5Ac) is one of the commonly distributed natural carbohydrates, and it is also a basic component of many glycoproteins, including glycopeptides and glycolipids, with a wide range of biological functions [12]. Moreover, the biochemical derivatives of Neu5Ac are also commonly used in the synthesis of medications [12]. Recent studies showed that Neu5Ac may participate in the pathogenesis of atherosclerosis by promoting immune inflammatory response [13, 14], regulating lipoprotein metabolism [15], enhancing insulin resistance [16], accelerating thrombosis formation [17], and stimulating vascular smooth muscle cell proliferation and apoptosis [18]. Moreover, inhibition of Neu5Ac via pharmacological inhibition of neuraminidase was associated with relieved myocardial injury in hypoxic cardiomyocytes and in an in vivo model of myocardial infarction (MI), which strongly demonstrates that Neu5Ac may be a novel target for improving myocardial ischemia [19]. However, the changes in serum Neu5Ac in patients with various subtypes of ACS, the association between serum Neu5Ac and coronary lesion characteristics, risk stratification, and the relationship between serum Neu5Ac and prognosis of ACS, to the best of our knowledge, have not been fully explored. Therefore, the aim our study was to investigate the role of serum Neu5Ac in a real-world ACS patient cohort.

## Methods

This study was a prospective cohort study including consecutive patients with symptoms of chest pain and dyspnea who received coronary angiography (CAG) in our department between October 2018 and July 2019. Written consent was obtained from each patient before enrollment in the study, and the protocol for the study was approved by the ethics committee of the First Affiliated Hospital of Bengbu Medical College (Approval No. BYYFY-2018KY23) before performance of the study.

### Patient inclusion criteria

Consecutive patient were included according to the following criteria: (1) patients had suspected ACS and received CAG within the study periods; and (2) data regarding baseline demographic factors, comorbidities, concurrent medications, CAG results, and biochemical parameters could be obtained, including age, gender, current smoking status, prevalence of hypertension, diabetes mellitus (DM), histories of atrial fibrillation (AF) and previous stroke, and total cholesterol (TC), triglyceride (TG), high-density lipoprotein cholesterol (HDL-C), low-density lipoprotein cholesterol (LDL-C), C-reactive protein (CRP), uric acid (UA), serum creatinine (SCr), lipoprotein (a) (Lp(a)); and (3) the serum level of Neu5Ac on admission was available. The exclusion criteria included severe hepatorenal insufficiency, hematopoietic system disease, severe infectious disease, tumor, and other malignant diseases. The diagnosis of the subtype of ACS, including UAP and MI, was judged by a group of experienced physicians based on the clinical manifestations, ECG, clinical biochemical indexes, and findings of CAG, according to current diagnosis and management guidelines for ACS [5–7]. The diagnoses of hypertension, DM, and AF were based on the medical data on admission and the self-reported medical histories of the patients. The definitions of MI were in accordance with the Third Universal Definition of Myocardial Infarction [20]. The Global Registry of Acute Coronary Events (GRACE) risk score was calculated at admission [21, 22]. The GRACE risk score is a specifically designed tool for risk stratification of ACS patients that has been confirmed to offer effective guidance for prognosis prediction and clinical treatment planning [1, 2]. The GRACE tool was developed through an international registry program for risk stratification of a broad spectrum of ACS patients and subsequently become a validated tool for risk stratification in over 42,000 patients with external validation in other cohorts. A simplified model was derived that predicts the risk of death based on eight variables (age, heart rate, systolic blood pressure, Killip class, creatinine concentration, elevated biomarkers of myocardial injury, cardiac arrest on admission, and ST-segment deviation). GRACE risk scores are categorized as “low-risk” (0 to 108), “intermediate-risk” (109 to 140), or “high-risk” (≥141).

### Measurement of serum Neu5Ac

For each patient, a 5-ml blood sample was taken after fasting for 12 h through the median cubital vein in the early morning after admission to the hospital. Blood samples were collected into tubes with anticoagulants of heparin sodium or EDTA. Samples with the anticoagulant of heparin sodium were used for measurement of biochemical parameters under a standard protocol of the Department of Clinical Laboratory of our hospital. For the measurement of Neu5Ac, the blood sample was drawn after the confirmed diagnosis of ACS for all patients. No fasting was required. After centrifugation, the blood sample was preserved in a freezer − 80 °C for subsequent analysis. The serum Neu5Ac concentration in each patient was detected by liquid chromatography-tandem mass spectrometry, as previously described [19, 23].

### Coronary angiography and severity evaluation

Coronary angiography was performed by an experienced cardiologist according to the conventional Judkins method. Radial artery puncture and rapamycin-coated stents were used in all patients. During the procedure, 1 ml of 1% lidocaine was used for local anesthesia, and 2000 U heparin and 200 μg nitroglycerin were injected through the sheath after the artery sheath was implanted. The results of CAG were judged and reported by a group of experienced physicians according to the current guidelines [24]. The decision regarding subsequent performance of percutaneous coronary intervention (PCI) was also made by the group of experienced physicians according to the current PCI guidelines [24]. The periprocedural medication and management were in accordance with the recommendations of current PCI guidelines [25], and dual antiplatelet therapies and statins were administered to all patients before the procedure. All patients received aspirin, ticagrelor, and rosuvastatin before the procedure, while ACEI/ARB and beta blockers were adjusted according to the blood pressure and heart rate of patients. Because patients with heart failure were excluded, aldosterone receptor antagonists were not used. The results of CAG and the processes of PCI were recorded in detail. The criteria for successful PCI were in accordance with international practice guidelines, which include residual stenosis ≤20% and TIMI grade 3 blood flow after the procedure. The severity of coronary lesions was quantitatively assessed using the Gensini score based on the distributions and degree of stenosis for the affected coronary arteries detected by CAG [14]. For patients who underwent stent implantation, dual antiplatelet therapies were maintained for at least 1 year, and other pharmacological therapies for CAD, including statins, were also administered according to the current guidelines [25].

### Follow-up and outcomes

The patients with a confirmed diagnosis of ACS were followed as outpatients by a group of trained cardiologists monthly after discharge. The incidence of major adverse cardiovascular events (MACEs), including a composite outcome of recurrent chest pain, heart failure, stroke, recurrent MI, bleeding, revascularization, stent thrombosis, stent restenosis, and death [26], was recorded.

### Statistical analysis

Continuous data were presented as means and standard deviations (SDs) if they were normally distributed; otherwise, medians and interquartile ranges (IQRs) were reported. Differences in the continuous data with normal distribution among groups were analyzed using analysis of variance (ANOVA), and for the non-normally distributed data, the non-parameter test was used. For the categorized data, numbers and percentiles were presented, and the χ^2^ test was used for comparison. Correlations between the serum Neu5Ac level and the clinical characteristics of ACS patients were analyzed via Spearman correlation analysis. Received operating characteristic (ROC) curve analysis was used to evaluate the potential predictive value of serum Neu5Ac for high-risk GRACE stratification and the diagnosis of MI. Multivariate logistic analyses were applied to determine the potential risk factors for MACEs during follow-up from among Neu5Ac, age, hypertension, smoking, DM, AF, previous history of stroke, and fasting blood glucose (FBG) at admission. A *p* < 0.05 was defined as statistically significant. The statistical analyses were performed using IBM SPSS Statistics 21.0 software.

## Results

### Patient characteristics at baseline

Overall, 766 patients with a suspected diagnosis of ACS who underwent CAG examination during the study period were included (mean age: 63.5 ± 10.5 years; 427 men and 388 women). According to the clinical manifestations, ECG features, serum troponin, and CAG findings, these patients were classified as UAP patients (*n* = 100, NSTEMI: 21, STEMI: 79), MI patients (n = 100) or non-CAD controls (*n* = 129). The baseline characteristics of the included patients are shown in Table 1. There were no significant differences in the proportions of patients with previous stroke or AF among the UAP, MI and control groups. Moreover, the admission serum levels of TC, LDL-C, Lp(a), D-dimer, and CRP were also not statistically different among these patients. However, patients with UAP and MI were older, more likely to be smokers, and more likely to have DM as compared to non-CAD controls (all *p* < 0.05). In addition, serum UA, TG, and FBG were higher in patients with MI than in those with UAP or non-CAD controls (all *p* < 0.05). Furthermore, compared to those with UAP, patients with MI were more likely to be male, have hypertension, and have a higher SCr at baseline (all *p* < 0.05). As for the severity of coronary lesions, comparisons between UAP and MI patients showed that MI patients had higher Gensini Scores (53.04 ± 35.96 vs 30.70 ± 28.61, *p* < 0.001; Table 1) and were more likely to have three-vessel coronary lesions (37% vs 30%, *p* = 0.001; Table 1).

**Table 1:** Baseline characteristics of ACS patients and controls

	UAP (*n* = 537)	MI (n = 100)	Control (n = 129)	F or χ^2^	p
Age (years)	64.96 ± 10.13	63.44 ± 10.51	57.43 ± 9.67*	28.960	0.000
Male (n, %)	299 (56)	78 (78)	51 (40)	33.833	0.000
Smoker (n, %)	89 (17)	21 (21)	13 (10)*	11.350	0.003
Hypertension (n, %)	300 (56)	67 (67)	63 (49)*	7.60	0.022
DM (n, %)	144 (27)	24 (24)	12 (9)*	17.763	0.000
AF (n, %)	21 (4)	5 (5)	6 (5)	0.337	0.845
Previous stroke (n, %)	49 (9)	6 (6)	9 (7)	1.460	0.482
FBG (mmol/L)	5.11 (4.54, 6.48)	5.96 (4.90, 8.23)**	4.95 (4.51, 5.75)	18.746	< 0.001
TC (mmol/L)	3.72 (3.03, 4.64)	3.50 (2.79, 5.01)	3.83 (3.08, 4.53)	0.363	0.834
TG (mmol/L)	1.34 (0.96, 1.94)	1.54 (1.02, 2.01)**	1.23 (0.86, 1.86)	6.807	0.003
HDL-C (mmol/L)	0.90 (0.75, 1.06)	0.83 (0.67, 1.00)	0.95 (0.79, 1.14)*	13.958	0.001
LDL-C (mmol/L)	2.01 (1.54, 2.62)	1.98 (1.39, 2.86)	2.08 (1.57, 2.56)	0.173	0.917
CRP (mg/L)	1.20 (0.50, 3.60)	2.30 (0.05, 6.18)	1.20 (0.60, 3.30)	1.158	0.560
UA (μmol/L)	301 (251, 364)	317 (258, 398)**	282 (243, 353)	8.223	0.016
SCr (μmol/L)	67 (60.5, 72)	70 (63.25, 86)	63 (56.5, 69)	34.574	< 0.001
D-dimer (mg/L)	0.27 (0.19, 0.45)	0.29 (0.19, 0.45)	0.23 (0.11, 0.39)	8.729	0.013
Lp(a) (mg/L)	225 (93.75, 387.75)	237 (90, 470)	217 (110, 453)	0.143	0.931
Neu5Ac (ng/ml)	227 (114, 312)	297 (220, 374) **	207 (114, 276)	17.505	< 0.001
Coronary lesions (n, %)					
Three vessels	205 (30)	56 (37)**	–	11.075	0.001
Two vessels	194 (26)	27 (23)	–	3.099	0.078
Single vessel	138 (44)	17 (40)	–	3.464	0.063
Gensini score	30.70 ± 28.61	53.04 ± 35.96**	–	6.876	0.000
CK-MB (U/L)	12.20 (0.00, 17.00)	40.62 (8.00, 42.00) **	9.49 (0.00, 14.00)	35.032	< 0.001
TnI (μg/L)	0.37 (0.00, 0.19)	4.28 (0.07, 3.76) **	0.36 (0.00, 0.35)	34.817	< 0.001

*, *p* < 0.05 compared to MI patients;

*, *p* < 0.05 compared to UAP patients;

*UAP* Unstable angina pectoris, *MI* Myocardial infarction, *DM* Diabetes mellitus, *AF* Atrial fibrillation, *FBG* Fasting blood glucose, *TC* Total cholesterol, *TG* Triglyceride, *HDL-C* High-density lipoprotein cholesterol, *LDL-C* Low-density lipoprotein cholesterol, *CRP* C-reactive protein, *UA* Uric acid, *SCr* Serum creatinine, *Lp(a)* Lipoprotein (a), *Neu5Ac* N-acetylneuraminic acid, *CK-MB* MB isoform of creatine kinase, *TnI* Troponin I

### Serum Neu5Ac in patients with ACS

**Fig. 1:**
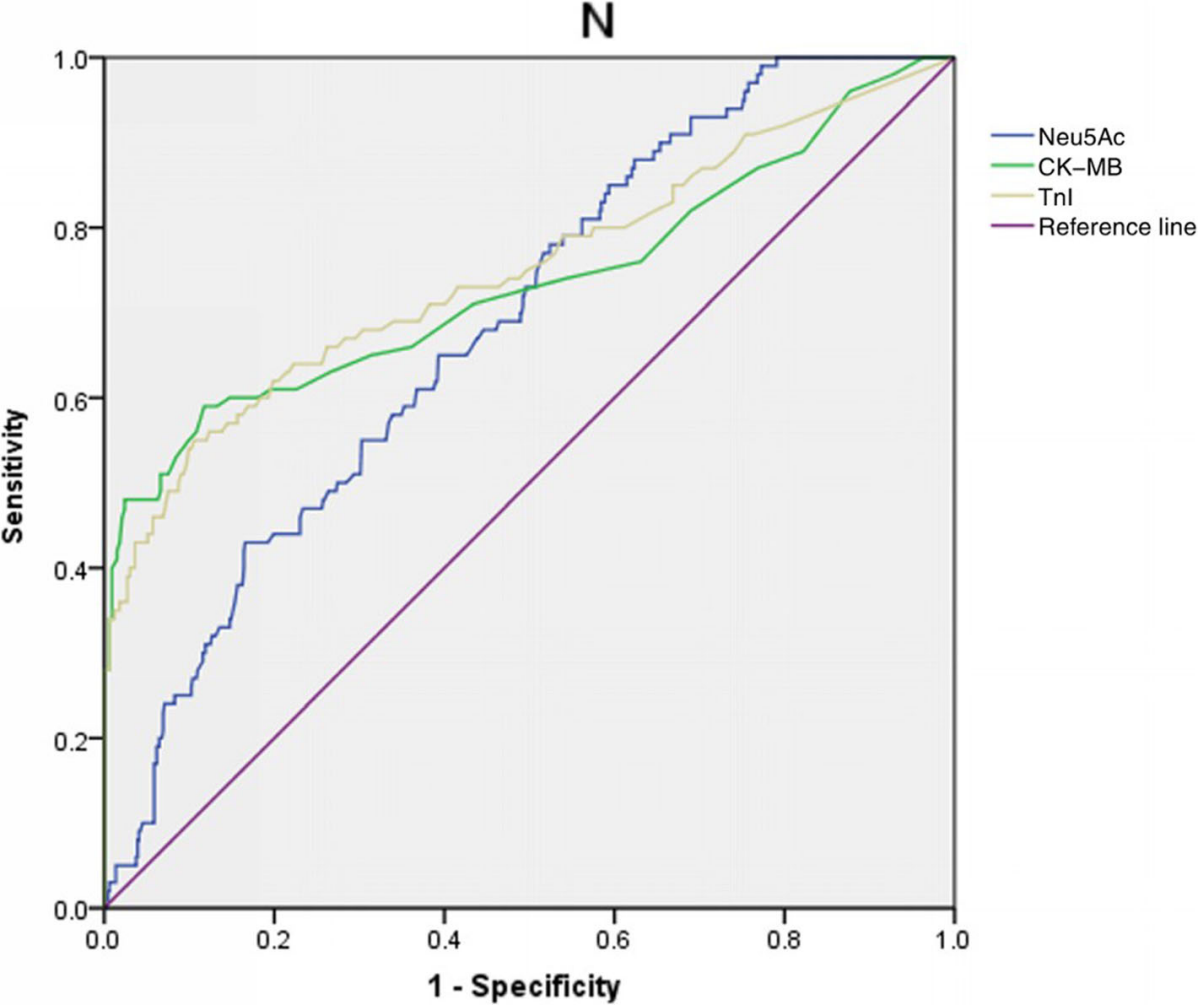
ROC curve analysis of the diagnostic efficacies of serum Neu5Ac, CK-MB, and TnI for MI in ACS patients

**Fig. 2:**
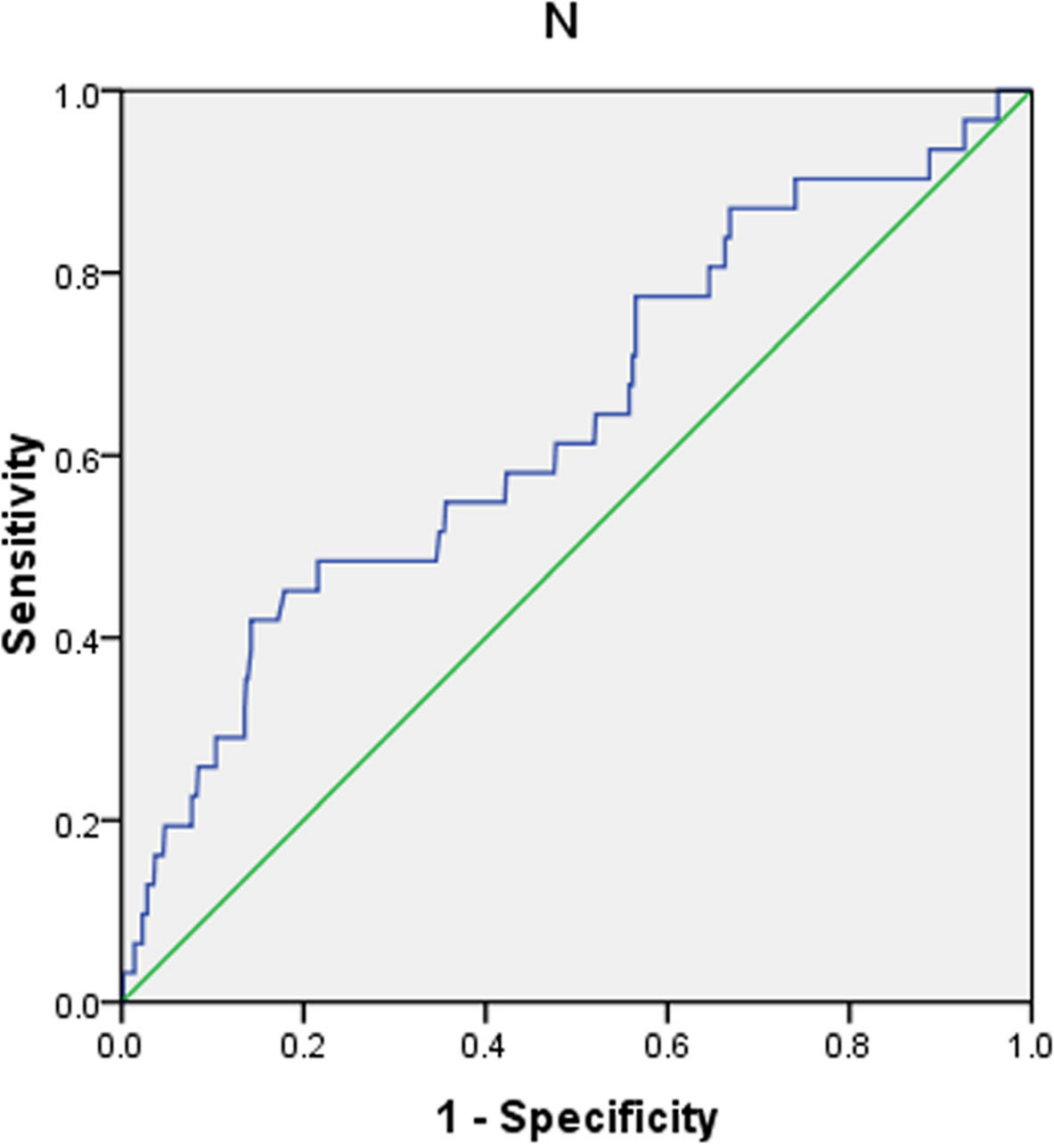
ROC curve analysis of the predictive value of serum N-acetylneuraminic acid on admission for high-risk GRACE score in ACS patients

Circulating Neu5Ac levels were compared among the groups and found to be significantly higher in patients with MI (median [1QR]: 297[220, 374] ng/ml) than in those with UAP (227 [114, 312] ng/ml) or in non-CAD controls (207 [114, 276] ng/ml; both *p* < 0.001; Table 1). Moreover, comparison of the serum Neu5Ac levels in patients with ST-elevation and non-ST elevation MI (STEMI vs NSTEMI) showed no statistically significant difference (302.24 ± 122.81 ng/ml vs 275.51vs 126.10 ng/ml, *p* = 0.389). Correlation analysis in patients with ACS showed that the serum level of Neu5Ac was positively correlated with age, the proportion of hypertension, serum UA, SCr, MB isoform of creatine kinase (CK-MB), and Gensini score (Table 2, all *p* < 0.001), but negatively correlated with the serum level of HDL-c (Table 2, *p* < 0.001). According to the GRACE risk stratification, 53, 270, and 341 ACS patients were considered as low-risk, intermediate-risk, and high-risk, respectively, and the serum Neu5Ac level differed significantly among the groups (low-risk: 214.59 ± 115.13 ng/ml; intermediate-risk: 253.29 ± 141.62 ng/ml; and high-risk: 299.51 ± 195.58 ng/ml; *p* < 0.001). From the ROC curve analysis, we found that with the optimal cut-off for serum Neu5Ac was 330.5 ng/ml. The ROC curves for Neu5Ac, CK-MB, and TnI are compared in Fig. 1. The ROC curve analysis showed that Neu5Ac, CK-MB, and TnI all offered diagnostic efficacy for MI, with AUC values of 0.69 (0.64–0.74), 0.74 (0.67–0.80), and 0.75 (0.69–0.81), respectively. There was no significant difference among the diagnostic efficacies of the three markers for MI (*p* > 0.05; Neu5Ac and CK-MB: z = 0.985, *p* = 0.324; Neu5Ac and TnI: z = 1.344, *p* = 0.179; CK-MB and TnI: z = 0.374, *p* = 0.709). It could be concluded that the diagnostic value of Neu5Ac for MI is not inferior to that of CK-MB and TnI. In addition, a higher serum Neu5Ac was associated with high-risk stratification based on GRACE score (AUC: 0.64 [0.53–0.75]) with a sensitivity of 41.9%, a specificity of 85.8%, and a cut-off of 351.5 ng/ml (Fig. 2).

**Table 2:** Correlation of serum Neu5Ac with clinical characteristics and severity of coronary lesions in ACS patients

	r	p
Age (years)	0.265	0.000
Hypertension (%)	0.262	0.000
UA (μmol/L)	0.312	0.000
SCr (μmol/L)	0.415	0.000
HDL-C (mmol/L)	−0.187	0.000
CK-MB (U/L)	0.232	0.000
Gensini score	0.283	0.000
FBG (mmol/L)	0.039	0.282
TC (mmol/L)	0.048	0.200
TG (mmol/L)	0.023	0.545
CRP (mg/L)	0.052	0.171
TnI (μg/L)	0.044	0.224

*Neu5Ac* N-acetylneuraminic acid, *ACS* Acute coronary syndrome, *UA* Uric acid, *SCr* Serum creatinine, *HDL-C* High-density lipoprotein cholesterol, *CK-MB* MB isoform of creatine kinase, *FBG* Fasting blood glucose, *TC* Total cholesterol, *TG* Triglyceride, *CRP* C-reactive protein, *TnI* Troponin I

### Serum Neu5Ac and the prognosis of ACS patients

During a median follow-up of 8 months, one patient with UAP was lost. The results showed that ACS patients with MACEs had higher baseline serum Neu5Ac levels compared to those without MACEs during follow-up (261.32 ± 170.40 ng/ml vs 226.87 ± 122.38 ng/ml, *p* = 0.009). The results of multivariate adjusted logistic analyses to determine the risk factors associated with the incidence of MACEs in patients with ACS are shown in Table 3. The results showed that the only independent predictor of MACEs in these patients was a higher serum Neu5Ac level (odds ratio [OR]: 1.003, 95% confidence interval [CI]: 1.002–1.005, *p* < 0.001), while other factors including age, hypertension, smoking, DM, AF, previous stroke, and FBG at baseline were not potential risk factors (all *p* > 0.1).

**Table 3:** Risk factors for MACEs in patients with ACS: results of multivariate logistic regression analysis

	B	Sx	Wald	OR (95% CI)	p
Neu5Ac (ng/ml)	0.003	0.001	16.351	1.003 (1.002–1.005)	0.000
Age (years)	0.011	0.011	0.936	1.011 (0.989–1.034)	0.333
Hypertension (%)	0.328	0.234	1.969	0.720 (0.456–1.139)	0.161
Smoking (%)	0.294	0.496	0.351	0.745 (0.282–1.971)	0.553
DM (%)	0.123	0.303	0.165	0.884 (0.488–1.602)	0.684
AF (%)	1.026	0.761	1.182	0.358 (0.081–1.591)	0.177
Previous stroke (%)	0.247	0.435	0.321	0.781 (0.333–1.834)	0.571
FBG (mmol/L)	0.013	0.052	0.059	1.013 (0.915–1.121)	0.807
CRP (mg/L)	0.005	0.007	0.516	1.005 (0.992–1.018)	0.473

*MACE* Major adverse cardiovascular events, *ACS* Acute coronary syndrome, *OR* Odds ratio, *CI* Confidence interval, *Neu5Ac* N-acetylneuraminic acid, *DM* Diabetes mellitus, *AF* Atrial fibrillation, *FBG* Fasting blood glucose, *CRP* C-reactive protein

## Discussion

In this prospective cohort study, by analyzing a real-world cohort of patients with suspected ACS who underwent CAG, we found that the serum Neu5Ac concentration was significantly higher in ACS patients than in controls with no CAD. Moreover, the serum level of Neu5Ac was even higher in patients with MI compared with that in patients with UAP, and the ROC curve analysis showed that an elevated serum Neu5Ac level is capable of predicting a diagnosis of MI. Moreover, an increased serum Neu5Ac level was closely correlated with conventional risk factors for CAD, including aging, hypertension, renal insufficiency, and low HDL-C. Importantly, a higher serum Neu5Ac level was correlated with more severe coronary lesions, high-risk on GRACE risk stratification, and poor prognosis in ACS patients. Taken together, the results of this pilot study demonstrated that serum Neu5Ac is associated with myocardial injury in ACS patients and may reflect the severity of coronary lesions and predict a poor prognosis in these patients. Future studies are needed to determine the molecular pathways mediating the role of Neu5Ac in acute myocardial ischemia and to clarify whether Neu5Ac is a biomarker only or a potential therapeutic target for myocardial injury caused by acute ischemia.

In recent years, the roles of metabolites in cardiovascular disease have attracted great attention. Comprehensive quantitative and qualitative analyses of various small molecule substances in patients with cardiovascular disease, such as CAD, have been confirmed to be helpful in elucidating the changes in endogenous metabolic substances within organisms after determination from the internal and external environment, helpful in identifying metabolic marker groups related to diseases, and useful for advancing the early diagnosis and treatment of the disease [27–30]. Neu5Ac, also known as sialic acid, is a naturally occurring amino glucosamine. It was originally isolated from bovine submandibular gland mucins. It belongs to the monosaccharide family with a 9-carbon main chain and a high degree of structural diversity. Recently, Neu5Ac has been proposed to have multiple biological functions. As a viral receptor, it is closely related to malignant transformation, cancer metastasis, invasion, loss of contact inhibition, cell adhesion reduction and antigenicity [31–33]. However, the mechanism of its role in CAD has not been fully understood. At present, it is suggested that plasma Neu5Ac may promote the formation of atherosclerosis through enhancing the inflammatory reaction, disrupting iron metabolism, promoting platelet thrombosis and other mechanisms [34, 35]. Moreover, the level of plasma Neu5Ac may be related to the severity of CHD, and the release of a large amount of intracellular and cell surface Neu5Ac into the blood at the early stage of MI is considered to be the main cause of the increased serum Neu5Ac level in these patients [19]. However, the relationships between serum Neu5Ac and the severity and prognosis of ACS have been rarely investigated.

In this study, we found that the serum level of Neu5Ac in patients with MI was significantly higher than that in patients with UAP or that in controls. We also divided the AMI group into STEMI and NSTEMI groups, and no significant difference in the serum Neu5Ac level was found between these two groups. These results suggest that serum Neu5Ac may be a metabolic marker of myocardial injury and necrosis, independent of the type of MI. Via ROC curve analysis, we found that the level of Neu5Ac in serum can assist in the diagnosis of acute MI, with a cut-off value of 330.5 ng/ml. Moreover, correlation analyses showed that the serum level of Neu5Ac was positively correlated with age, hypertension, CKMB, Gensini score, UA and SCr, and negatively correlated with the level of HDL-C. All of these factors have been confirmed to be closely correlated with the degree of myocardial injury. Taken together, these findings demonstrate that the increased level of serum Neu5Ac in MI patients might reflect the degree of cardiac necrosis, metabolism and the severity of coronary artery disease. Therefore, measurement of serum Neu5Ac may be of significance for the early identification of patients at high risk for the development of MI.

In a previous study, Zhang et al. [19] analyzed a large number of plasma samples using non-targeted metabolomics and found that Neu5Ac can activate the Rho/ROCK signaling pathway by combining RhoA and Cdc42 and thereby caused myocardial injury both in vitro and in vivo. Rho kinase has two isomers, Rock1 and Rock2, which are expressed in vascular smooth muscle and heart. Activation of the Rho/Rho kinase signaling pathway through different pathways leads to phosphorylation of myosin light chain (MLC) and aggregation of integrin, which results in increased permeability of endothelial cells. The subsequent molecular mechanisms for upregulation of Neu5Ac and activation of Rho/Rho kinase signaling may include monocyte/macrophage migration, transportation of oxidized low-density lipoprotein, endothelial dysfunction, as well as phenotype switch and proliferation of vascular smooth muscle cells, which all contribute to the pathogenesis of atherosclerosis.

Current diagnosis of ACS is based on CKMB and troponin combined with the symptoms, ECG, and coronary angiography findings of patients. However, it has been suggested that these parameters may not be adequate for the guidance of individualized treatment. Interestingly, clinical studies have shown that the recurrence rate of cardiovascular events in patients diagnosed with influenza and treated with oseltamivir or other anti-influenza drugs is significantly lower than that among patients not treated with anti-influenza drugs [36, 37]. Notably, the active metabolites of oseltamivir play a role by inhibiting neuraminidase-1. Therefore, drugs that inhibit neuraminidase-1 may be useful for protecting myocardial cells and heart tissue from myocardial injury in the future, representing a new intervention for CAD treatment. These findings highlight the potential therapeutic significance of Neu5Ac metabolism as a novel target for the treatment of myocardial ischemia. In our study, during the follow-up (5–14 months) after discharge, we found that serum Neu5Ac levels in patients with MACEs were significantly higher than those in patients who did not experience MACEs, and our binary correlation analysis showed that the serum level of Neu5Ac was an independent risk factor for MACEs. These findings suggest that the serum level of Neu5Ac is related to the clinical prognosis of ACS patients, and therapeutics that reduce the serum Neu5Ac level may also reduce the incidence of MACEs in these patients. Whether pharmacological interventions to inhibit Neu5Ac can improve the prognosis of ACS patients will need to be determined in future clinical trials.

Despite of the potential strengths of our study as the first real-world study to elucidate the role of Neu5Ac in ACS patients, our study also has limitations. First, due to the limited sample size, we were unable to determine whether the significance and potential prognostic role of Neu5Ac change according to patient or study characteristics, such as age, gender, ethnicity, DM, or concurrent medications. Moreover, although some potential confounding factors were adjusted in the analysis of the association between elevated serum Neu5Ac and poor outcomes in ACS patients, we could not exclude the possibility that residual factors may confound the association, such as patients’ nutritional status and the administration of medications that influence Neu5Ac metabolism during follow-up. Finally, as an observational study, we were unable to determine whether the relationships between an elevated Neu5Ac level and the severity and prognosis of ACS are causative. In-depth experimental studies and randomized controlled trials are needed to further characterize these relationships.

## Conclusions

In conclusion, the results of our real-world study demonstrated that serum Neu5Ac is associated with myocardial injury in ACS patients and that it may reflect the severity of coronary lesions, correlate with high-risk GRACE risk stratification, and predict poor prognosis in these patients. Thus, serum Neu5Ac may become a biomarker for the diagnosis and risk stratification of ACS patients, and targeted Neu5Ac inhibition may represent a novel therapeutic strategy for CAD. Studies are needed to further our understanding of the mechanisms and therapeutic potential of treatments targeting Neu5Ac in patients with acute myocardial ischemia.

## Data Availability

The datasets generated and analyzed in the present study are available from the corresponding author upon reasonable request.

## Abbreviations

ACS: Acute coronary syndrome
CAD: Coronary artery disease
STEMI: ST-segment elevation myocardial infarction
NSTE-ACS: Non ST-segment elevation ACS
ECG: Electrocardiography
PCI: Percutaneous coronary intervention
MI: Myocardial infarction
CAG: Coronary angiography
DM: Diabetes mellitus
AF: Atrial fibrillation
TC: Total cholesterol
TG: Triglyceride
HDL-C: High-density lipoprotein cholesterol
LDL-C: Low-density lipoprotein cholesterol
CRP: C-reactive protein
UA: Uric acid
SCr: Serum creatinine
MACEs: Major adverse cardiovascular events
SDs: Standard deviations
IQRs: Interquartile ranges
ROC: Received operating characteristic
FBG: Fasting blood glucose
MLC: Myosin light chain
